# Effect of High-Flow Nasal Cannula for Hypoxemia Following Sun's Procedure in Acute Aortic Dissection Type a Patients

**DOI:** 10.3389/fsurg.2021.630624

**Published:** 2021-05-07

**Authors:** ChaoJun Yan, Jianrong Zhang, Yi Wu, Jie Yao, Jun Li, Xianpu Zhang, Yongbo Cheng, Xin Liu, Jianguang Yi, Deqin Lin, Sanjiu Yu, Mei Guo, Liuhong Lu, Wei Cheng, Ping He

**Affiliations:** ^1^Cardiac Surgery Department, Southwest Hospital, Army Medical University, Chongqing, China; ^2^Cardiac Surgery Department, Three Gorges Hospital, Chongqing University, Chongqing, China; ^3^Institute of Digital Medicine, College of Biomedical Engineering and Medical Imaging, Army Medical University, Chongqing, China

**Keywords:** HFNC, hypoxemia, Sun's procedure, lung volume loss, 3D reconstruction of CT images

## Abstract

**Background:** Patients with acute aortic dissection type A (AADA) often have hypoxemia (partial pressure of oxygen [PaO_2_]/fraction of inspired oxygen [FiO_2_] <300 mmHg) before weaning in the intensive care unit (ICU). This study compared the efficacy of high-flow nasal cannula (HFNC) with that of conventional oxygen therapy (COT) in patients with AADA following Sun's procedure.

**Methods:** The medical records of 87 adult patients with AADA who underwent Sun's procedure and met the inclusion criteria (PaO_2_/FiO_2_ <300 mmHg before weaning) were retrospectively analyzed. After surgery, 41 patients were treated with HFNC and 46 were treated with COT. The oxygenation level, FiO_2_, partial pressure of carbon dioxide, heart rate, respiratory rate, subjective discomfort, and reintubation rate were recorded. The difference in lung volume loss between the HFNC and COT groups was assessed using the radiological atelectasis score (chest radiograph) or calculated from three-dimensional (3D) reconstructed computed tomography (CT) images.

**Results:** From day 1 to day 5 after weaning, there was no significant difference in PaO_2_/FiO_2_ between the HFNC and COT groups, although the FiO_2_ was significantly lower in the HFNC group than in the COT group (*P* < 0.05). Further studies indicated that the percentage of lung volume loss (pleural effusion and/or pulmonary atelectasis) by 3D reconstruction of CT images at 4–8 days post-operation was significantly lower in the HFNC group (*P* < 0.05). The subjective experience of breathing discomfort, reintubation rate, and length of stay in the ICU were significantly reduced in the HFNC group (*P* < 0.05). There was no significant difference in readmission to the ICU and in-hospital mortality between the two groups.

**Conclusions:** HFNC can be used as an effective oxygen therapy for AADA patients with hypoxemia after Sun's procedure.

## Introduction

Acute aortic dissection type A (AADA) is a fatal disease with a mortality rate of 1–2% per hour on the first day and ~50% mortality within the first week (1). Surgeries are life-saving measures for most patients with AADA. However, these patients have higher rates of postoperative hypoxemia (30–50%) than for other cardiac surgeries (valvular heart surgery and others) (2). Hypoxemia often leads to prolonged postoperative mechanical ventilation and intensive care unit (ICU) stay, as well as increased postoperative mortality (3). In addition, pulmonary atelectasis is found in up to 90% of patients after cardiac surgery (4). Postoperative atelectasis is associated with prolonged oxygen therapy and delayed recovery.

Effective oxygen therapy is one of the most important priorities for patients with postoperative AADA. High-flow nasal cannula (HFNC) is widely used in patients after cardiac surgery, and its effect on hypoxemia in Sun's procedure has not been reported. HFNC has been applied immediately after cardiothoracic surgery at several institutions, and has been proven to significantly improve patient comfort and to reduce the need for increased respiratory support (5). Compared with conventional oxygen therapy (COT), HFNC has the advantages of being easily tolerated, enabling natural eating and drinking, and does not affect cough and sputum production. Thus, it is considered beneficial for respiratory treatment and can even replace COT (6, 7). Corley et al. observed the beneficial effects of HFNC, such as a more pronounced increase in end-expiratory lung volume in a cardiac surgical population (8). Whether using HFNC in patients with postoperative AADA is better than COT is still to be determined. Therefore, in this study, we retrospectively analyzed patients who only underwent Sun's procedure, one of the surgical methods of AADA, and evaluated the effect of HFNC following Sun's procedure.

## Materials and Methods

### Patient Selection

**Figure 1:**
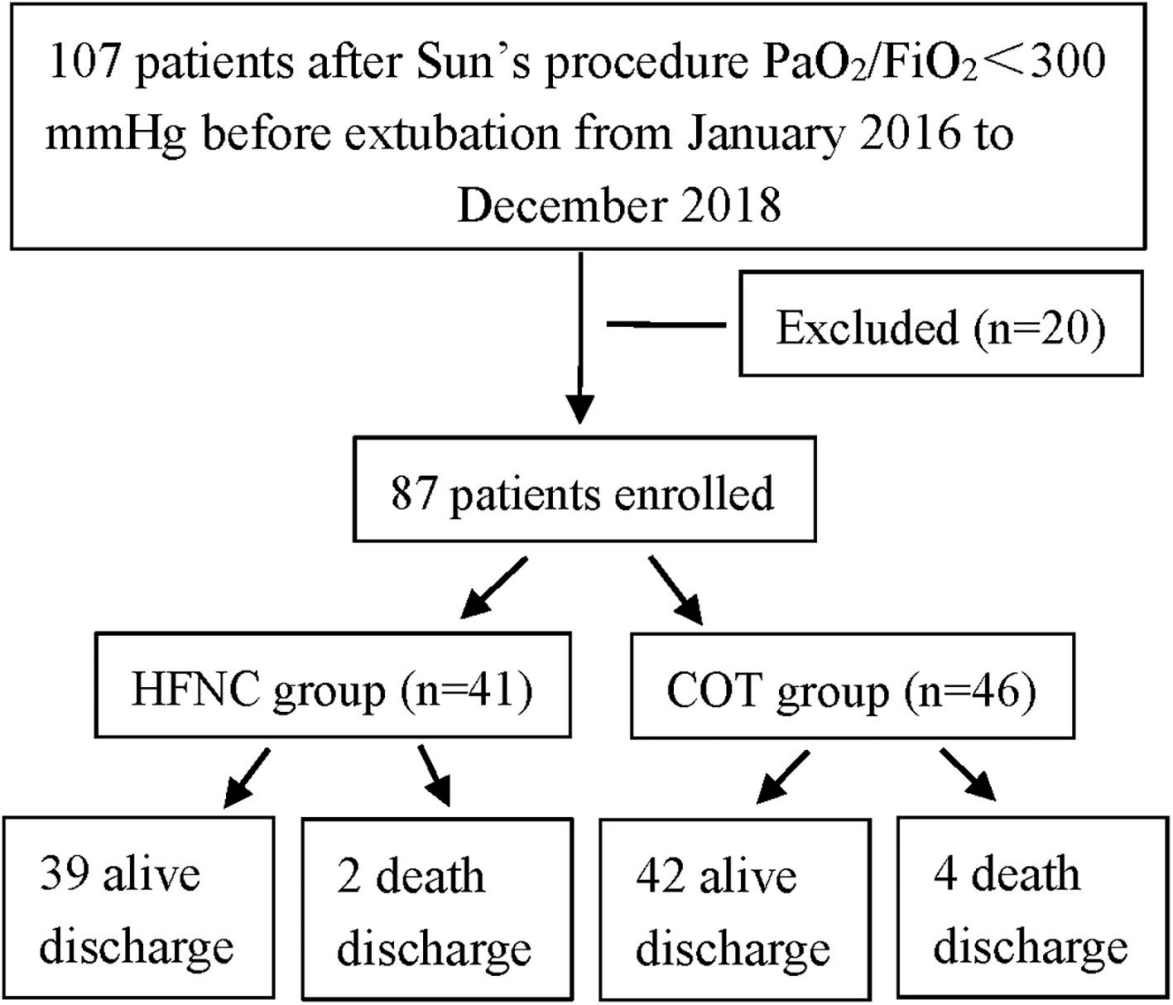
Patient flow chart.

We retrospectively analyzed adult patients who underwent Sun's procedures in the Departments of Cardiac Surgery of Southwest Hospital and Three Gorges Hospital between January 2016 and December 2018. In these hospitals, COT was used in all patients before July 2017, and HNFC was used thereafter (Figure 1). Other therapies, including diuretics in the ICU, did not differ between the two groups. Ethical approval was granted by the Institutional Human Research Ethics Committee (KY 2019159 and KY 2021047).

### Inclusion and Exclusion Criteria

Patients (aged > 18 years) were included if they (1) underwent Sun's procedure, (2) had partial pressure of oxygen (PaO_2_)/fraction of inspired oxygen (FiO_2_) <300 mmHg before weaning, and (3) showed a percentage of lung volume loss <5% on preoperative three-dimensional (3D) computed tomography (CT) before surgery. Patients were excluded if they (1) underwent any surgery (including valve replacement and coronary artery bypass grafting) other than the Sun's procedure; (2) had mechanical ventilation for >5 days; (3) died within 24 h after surgery; (4) had low swallowing and cough reflex; (5) had severe postoperative complications, such as coma, cardiogenic shock, gastrointestinal ischemia, cardiac arrest history, and multiple organ dysfunctions; and (6) planned to use non-invasive ventilation after weaning.

### Postoperative Treatments

Patients in the COT group received standard respiratory care under the direction of the treating ICU consultant with oxygen delivered at 2–4 L/min *via* nasal cannula, 6 L/min *via* simple face mask or oxygen masks titrated to maintain an oxygen saturation (SpO2) of ≥93–95%, or as consultant-directed by the bedside doctor. Patients in the HFNC group received 20–60 L/min of oxygen using the AIRVO2 nasal high-flow device from Fisher & Paykel Healthcare (PT101AZ), New Zealand. In both groups, FiO_2_ was adjusted to maintain a PaO_2_ of >60 mmHg for patients in the ICU or a SpO_2_% of ≥93–95% for patients in the general ward (9, 10). If the ideal oxygenation level was not reached, the inspiratory flow and oxygen concentration gradually increased. Patients were subjected to intubation again if hypoxemia was not properly controlled, which included an increased respiratory rate (RR), worsening gas exchange, acute respiratory failure, and patient intolerance as indicated by shortness of breath, RR > 30 breaths/min after oxygen therapy, respiratory muscle fatigue, PaO_2_ <60 mmHg, PaCO_2_ > 50 mmHg, or pH <7.30.

Patients on HFNC or COT in the ICU were monitored for arterial blood gas. Patients who were in good general condition and continuously accepted HFNC or COT after being transferred to the general ward from the ICU were subjected to telemetry electrocardiography to monitor the heart rate (HR) and RR.

### Assessment of Lung Volume Loss by RAS and 3D Reconstruction of CT Images

All patients underwent multidetector-row CT before the surgery to evaluate the loss of lung volume (11). Chest radiography was performed on days 1–5 after weaning and scored for atelectasis severity using a radiological atelectasis score (RAS) by radiologists (12).

Within 4–8 days after the operation, the patients in the two groups were reexamined with chest CT. Using Amira software tools (http://www.amiravis.com, version 5.3.3), the normal lung, atelectasis (or lung consolidation), and pleural effusion were segmented and 3D reconstructed. The 3D model was then smoothed and simplified. Lung volume loss caused by atelectasis (or lung consolidation) and pleural effusion were measured and compared between the two groups.

### Data Collection

Clinical data of patients who were weaned from mechanical ventilation were collected. Baseline demographic and clinical data were collected before weaning. The patients' subjective feelings of throat/nose and airway dryness were examined by doctors each day during oxygen therapy. After weaning, PaO_2_ of arterial blood gases, FiO_2_, HR, and RR were recorded from weaning to the end of oxygen therapy. The daily value was derived from the average of the nursing records over the course of a day. A reduction in the percentage of lung volume loss at 1–5 days after surgery and within 4–8 days during postoperative oxygen therapy was calculated by either RAS or 3D reconstruction of CT images.

### Statistical Analyses

Data are expressed as means ± standard deviations, frequencies, or percentages. Categorical variables were compared using the chi-square test or Fisher's exact test, and continuous variables were compared using the dependent or *t* test. Statistical significance was defined as *P* < 0.05. All statistical analyses were performed using commercially available statistical software (SPSS v22.0). GraphPad Prism 6 was used for drawing.

## Results

### Baseline Characteristics

A total of 107 patients were admitted with AADA, of whom, 20 were excluded. The remaining 87 patients who underwent Sun's procedure were enrolled in this study. Among them, 41 received HFNC, and 46 received COT (Figure 1). In addition, there were no significant differences between the two groups at baseline (Table 1).

**Table 1:** Baseline patient demographic and clinical characteristics of the HFNC and COT groups.

**Factors**	**COT (*n* = 46)**	**HFNC (*n* = 41)**	***P***
Age	49.51 ± 11.14	52.11 ± 11.00	0.308
Gender (male, %)	32 (69.57%)	27 (65.85%)	0.711
BMI (kg/m2)	21.63 ± 2.99	21.14 ± 2.27	0.424
Smoking history	24 (52.17%)	19 (46.34%)	0.587
The operation time (min)	455.34 ± 67.91	464.22 ± 76.09	0.590
CBP duration (min)	247.86 ± 39.16	257.38 ± 55.79	0.383
Aortic occlusion time (min)	124.90 ± 22.11	120.63 ± 25.55	0.435
DHCA (min)	40.67 ± 9.83	35.63 ± 11.85	0.450
RBC intraoperative (ml)	900 (800–1,300)	1070 (600–1,675)	0.689
Blood loss in first 12 h (ml)	600 (400–900)	550 (400–800)	0.521
Duration of intubation (h)	32 (25–67)	37.5 (26–114.7)	0.292
**Oxygenation impairment classify**			
Mild	30 (65.22%)	19 (46.34%)	0.103
Moderate	13 (28.26%)	18 (43.90%)	
Severe	3 (6.52%)	4 (9.76%)	

*Data are expressed as number (%), mean ± standard deviation, or median (interquartile range), depending on variable distribution. BMI, body mass index; RR, respiratory rate; DHCA, deep hypothermia circulatory arrest*.

### Comparison of the Clinical Outcomes Between the HFNC and COT Groups

The proportion of patients with sore throat/nose and dry airway was significantly lower in the HFNC group than in the COT group (23.91 vs. 7.32%, *P* = 0.044), and the rate of reintubation and length of stay in the ICU were significantly lower in the HFNC group than in the COT group [17.39 vs. 2.44%, *P* = 0.032, 94.5 (74.5–149.25) vs. 76 (53–130), *P* = 0.031]. There was no significant difference in readmission to the ICU (10.87 vs. 4.88%, *P* = 0.439) and mortality (8.70 vs. 4.88%, *P* = 0.680) (Table 2).

**Table 2:** Comparison of the outcome event between the HFNC and COT groups.

**Factors**	**COT(*n* = 46)**	**HFNC(*n* = 41)**	***P***
Sore throat/nose, airway dry	11 (23.91%)	3 (7.32%)	0.044
Reintubation	8 (17.39%)	1 (2.44%)	0.032
readmitted to ICU	5 (10.87%)	2 (4.88%)	0.439
In-hospital mortality rate	4 (8.70%)	2 (4.88%)	0.680
ICU length of stay (h)	94.5 (74.5–149.25)	76 (53–130)	0.031

### Comparison of Variables at Different Time Points Between the HFNC and COT Groups

**Figure 2:**
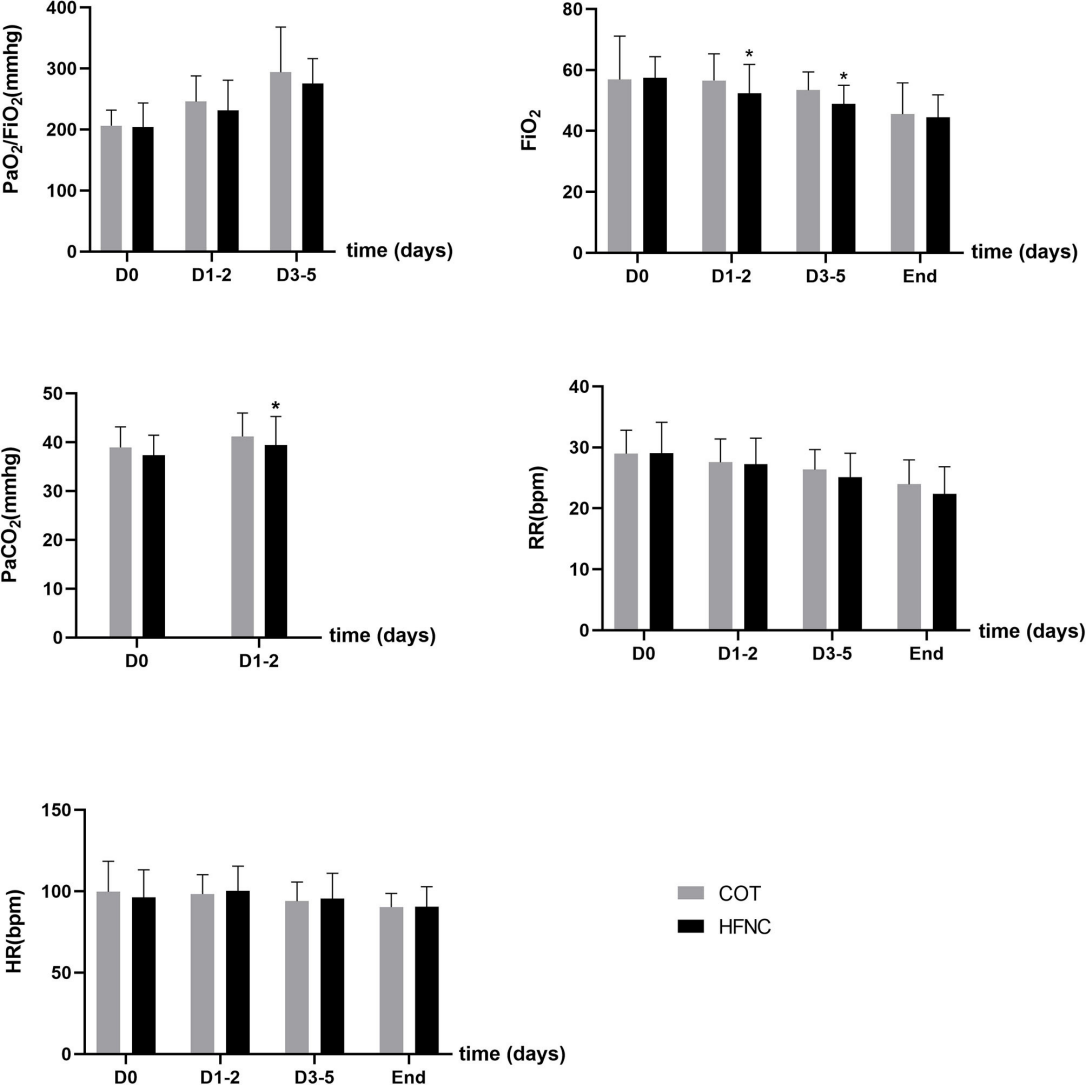
Changes in clinical parameters in oxygen therapy treatment. *P < 0.05.

From day 1 to day 5 after weaning, there was no significant difference in PaO_2_/FiO_2_ between the HFNC group and COT group (203.98 ± 39.39 vs. 206.17 ± 25.75 on the day of surgery, 231.39 ± 49.50 vs. 245.67 ± 42.08 in 1–2 days, 275.09 ± 40.97 vs. 293.78 ± 74.03 in 3–5 days) (*P* > 0.05), although FiO_2_ of the HFNC group was significantly lower than that of the COT group (52.29 ± 9.56 vs. 56.59 ± 8.69 in 1–2 days, 48.80 ± 6.19 vs. 53.35 ± 6.00 in 3–5 days) (*P* < 0.05). Although patients in the HFNC group had a lower RR than those in the COT group, this difference was not significant. There was also no statistically significant difference in HR and PaCO_2_ between the two groups (Figure 2).

### Loss of Lung Volume Measured by Either RAS Score or 3D Reconstruction of CT Images

**Figure 3:**
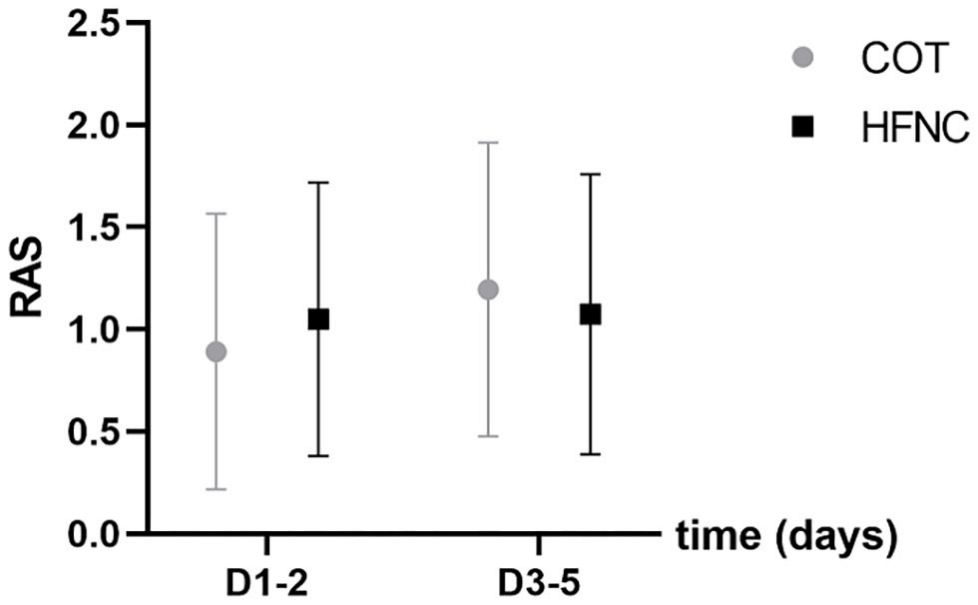
Comparison of the atelectasis score (RAS) between the HFNC and COT groups. Pre refers to pre-operation.

**Figure 4:**
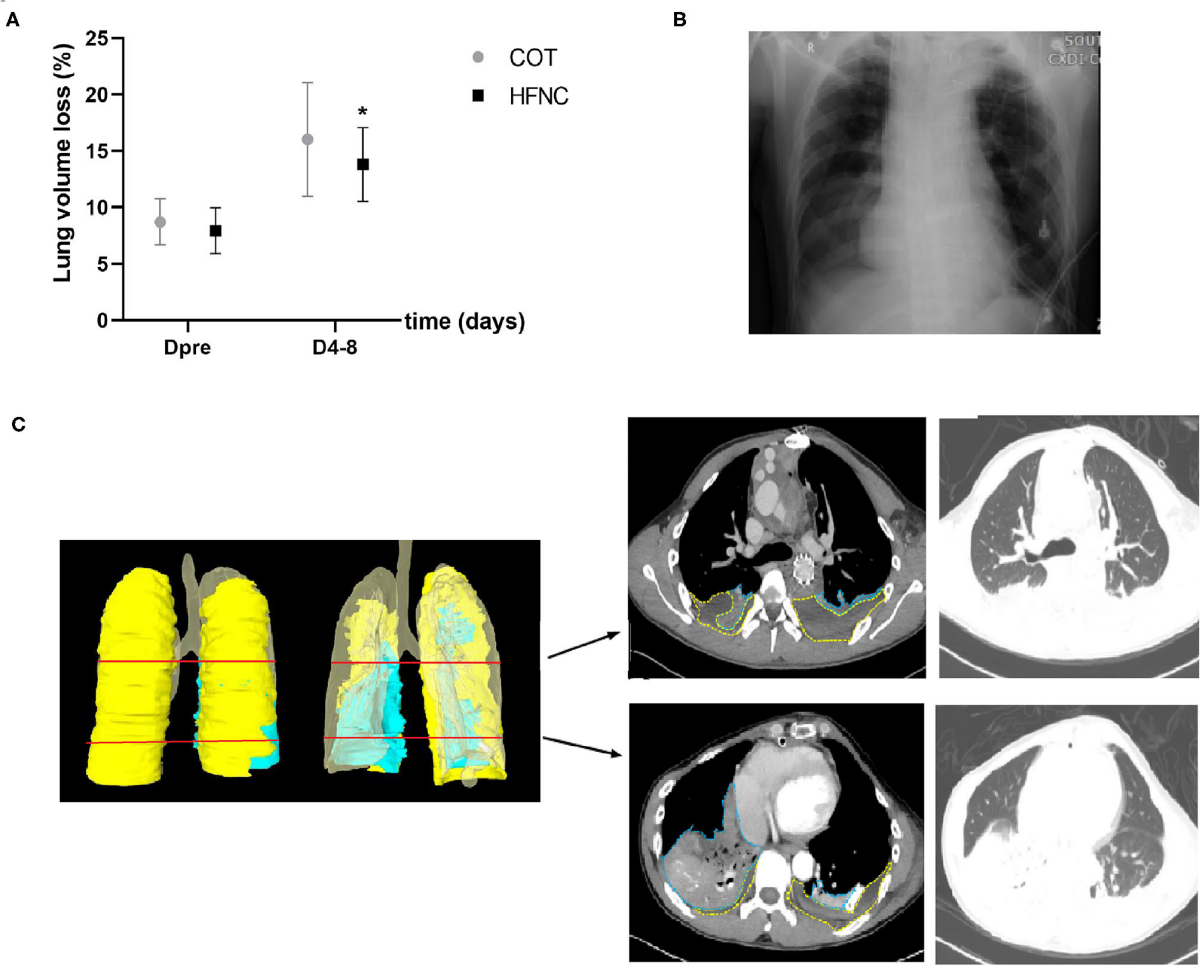
Comparison of lung volume loss between the HFNC and COT groups by reconstructing images of 3-dimensional computed tomography. **(A)** Lung volume loss; Pre refers to Pre-operation. **(B)** Chest radiographs of the patient. **(C)** 3-dimensional image of thoracic and CT images of the same patient on the same day. Light blue is atelectasis (AC), light yellow represents pleural effusion (PE), the clear area represents the lung.

There was no significant difference between the two groups in lung volume loss before surgery (assessed by 3D reconstruction of CT images) and 1–5 days post-operation (assessed by chest X-ray, RAS) (*P* > 0.05). Furthermore, we used 3D reconstruction of CT images to re-calculate postoperative lung volume and found that the percentage of lung volume loss in the HFNC group was significantly smaller than that in the COT group within 4–8 days post-operation (Figures 3, 4).

## Discussion

AADA treatment involves multiple therapeutic modalities, such as total aortic arch replacement and hybrid frozen elephant trunk (FET) procedures. Among them, Sun's procedure has been widely utilized in China and appears to be an effective modality with an in-hospital mortality of 4.73% and a re-operation rate of 2.34% (13). The procedure includes total aortic arch replacement using a tetrafurcated graft with implantation of a specially designed frozen elephant trunk (Cronus® in the descending aorta (14, 15). However, 30–50% of postoperative AADA patients suffer from hypoxemia (16). To reduce the difference in postoperative hypoxemia caused by the surgical method in these patients, we chose AADA patients after Sun's procedure, which has been performed more often in China recently, as the research object.

Oxygen therapy after Sun's procedure is a common clinical problem. Previous reports have illustrated the effectiveness of HFNC in cardiac surgery (5, 8). However, failed HFNC in patients with pulmonary complications can lead to delayed intubation and increased morbidity and mortality (17). Although the safety and effectiveness of HFNC have attracted increasing attention, the results have been inconsistent. Therefore, in this study, only patients with Stanford type A AAD who had normal heart function by ultrasonic cardiogram and underwent Sun's procedure were enrolled to ensure the consistency of the samples.

Since the standard nasal cannula or mask is unable to provide a flow that exceeds the patient' s inspiratory volume, a mixture of supplied oxygen and entrained air is used as the inhaled gas. When patients were given a standard mask or nasal cannula for oxygen inhalation at admission, the fraction of delivered oxygen was less than that of the FiO_2_. In contrast, in the HFNC treatment, the provided flow matched the patients' inspiratory volumes. Thus, the actual fraction of inhaled oxygen is very close to that of the FiO_2_ and is easy to titrate. Unlike HFNC, oxygen masks or nasal cannulas in COT cannot provide a flow greater than the patients' inhalation volume, and the inspiratory flow is a mixture of supplied oxygen with entrained air. Therefore, when using a mask or nasal cannula in the COT, the oxygen delivered is less than the FiO_2_. In clinical COT treatment, to achieve similar oxygenation, it is necessary to increase the oxygen concentration and oxygen flow. HFNC can provide low FiO_2_, while a low-flow COT system can provide up to 50–60% of FiO_2_ (18). Because HFNC delivers gas at a flow rate exceeding the patients' maximum inspiratory rate, the final oxygen concentration delivered to patients is equivalent to that of the FiO_2_ (19). In this study, we also found that at similar PaO_2_/FiO_2_ to maintain oxygenation of patients, the FiO_2_ of patients in the COT group was significantly increased compared with that of the HFNC group.

A systematic review and meta-analysis of randomized controlled trials evaluating the effect of HFNC treatment on the need for reintubation in adult patients showed that HFNC treatment does not delay reintubation, and the oxygenation level is the same as that of non-invasive ventilation (20). Parke et al. (10) treated 340 patients after cardiac surgery (valve replacement and CABG, etc.) using both HFNC and COT and found no statistically significant difference in pH and PaCO_2_ between the two groups. Compared with that in the HFNC group, SpO_2_/FiO_2_ of patients in the COT group was significantly higher at 30 min, 4 h, 1 d, and 2 days after weaning. In this study, it was also found that PaO_2_/FiO_2_ in the COT group was higher than in the HFNC group, but the difference was not significant.

Parke et al. (10) and Corley et al. (21) found no difference in atelectasis based on RAS between the HFNC and COT groups. Our study also revealed that HFNC following Sun's procedure in patients does not improve atelectasis based on RAS when compared to COT. Some AADA patients who underwent Sun's procedure cannot be simply diagnosed with acute respiratory distress syndrome because they do not have a typical radioactive lung infiltration (22, 23). Therefore, in Tatsuishi's study using more accurate CT measurements, the use of HFNC therapy was associated with a reduction in both postoperative atelectasis in patients who underwent off-pump coronary artery bypass graft surgery. Landquist et al. (24) reported that atelectasis occurred in 87% of patients who underwent anesthesia or mechanical ventilation. Zochios et al. (5) reported that a PEEP of 4.0 cmH_2_O was observed at 60 L/min HFNC. Corley et al. investigated the effects of HFNC on end-expiratory lung volume (EELV), and increases in EELV were significantly influenced by BMI (12). The use of positive pressure ventilation or PEEP can prevent atelectasis, reduce a certain degree of pulmonary edema, and improve heart function and continuous positive airway pressure (CPAP) (25). We observed that the percentage of lung volume loss was significantly different in CT images during the oxygen therapy stage (*P* < 0.05). Our study also suggested that oxygenation was similar in the HFNC and COT groups, which may be related to the reduction in lung volume loss. Postoperative lung volume loss was associated with long-term oxygen therapy and delayed recovery, and the rate of reintubation and length of stay in the ICU were significantly lower in the HFNC group than in the COT group.

Previous studies on 105 patients with PaO_2_/FiO_2_ <300 mmHg before weaning found that HFNC therapy had a better improving effect on airway dryness symptoms than Venturi under the same FiO_2_ (26). Previous studies (12, 27) have demonstrated reductions in both RR and dyspnea with HFNC. In our study, the subjective discomfort of airway dryness in the HFNC group was significantly lower than that in the COT group (*P* < 0.05).

Our study has several limitations. First, this was a single-center retrospective study with a small sample size and limited population. Retrospective data collection and evaluation may have certain unavoidable biases. Therefore, our results need to be further verified in a prospective, multicenter, randomized controlled study with a large sample size. Second, the time points of CT evaluation after oxygen therapy are not consistent, which could result in certain deviations in data collection and result evaluation, and need to be carefully selected in future studies. Third, increasing the size of patients with different oxygenation index levels may lead to more meaningful results.

In summary, this study showed that for AADA patients who underwent Sun's procedure, HFNC treatment at post-weaning could reduce the loss of lung volume and inhaled oxygen concentration and improve subjective comfort. However, further multicenter trials are necessary to clarify the effectiveness and mechanism of Sun's procedure in these patients.

## Data Availability Statement

The raw data supporting the conclusions of this article will be made available by the authors, without undue reservation.

## Ethics Statement

The studies involving human participants were reviewed and approved by the Ethical Committee of Southwest Hospital (the number/ID of the approval is KY 2019159). The patients/participants provided their written informed consent to participate in this study.

## Author Contributions

PH: conception and design. PH and WC: administrative support. PH, JZ, YC, and YW: provision of study materials or patients and data analysis and interpretation. PH, JZ, YC, and JYa: collection and assembly of data. All authors: manuscript writing and final approval of manuscript.

## Conflict of Interest

The authors declare that the research was conducted in the absence of any commercial or financial relationships that could be construed as a potential conflict of interest.
